# Alteration in Serum Levels of Tumor Necrosis Factor Alpha is associated with Histopathologic Progression of Gastric Cancer

**DOI:** 10.52547/ibj.3847

**Published:** 2022-12-21

**Authors:** Mehdi Alikhani, Maryam Esmaeili, Mohammad Tashakoripour, Mohammad Ali Mohagheghi, Mahmoud Eshagh Hosseini, Eliette Touati, Massoud Vosough, Marjan Mohammadi

**Affiliations:** 1 HPGC Research Group, Medical Biotechnology Department, Biotechnology Research Center, Pasteur Institute of Iran, Tehran, Iran;; 2 Gastroenterology Department, Amiralam Hospital, Tehran University of Medical Sciences, Tehran, Iran;; 3 Cancer Institute, Tehran University of Medical Sciences, Tehran, Iran;; 4 Institut Pasteur, Unit of Helicobacter Pathogenesis, CNRS UMR2001, 25-28 Rue du Dr Roux, 75724 Paris Cedex 15, France;; 5 Department of Regenerative Medicine, Cell Science Research Center, Royan Institute for Stem Cell Biology and Technology, ACECR, Tehran, Iran

**Keywords:** Inflammation, Interleukin-8, cytokines, Tumor necrosis factor-alpha

## Abstract

**Background:**

The role of inflammatory cytokines, such as TNF-α and IL-8, in gastric carcinogenesis has been investigated, but their impact remains to be further elucidated.

**Methods:**

In this study, we measured the serum concentrations of these cytokines and *H. pylori* serostatus in dyspeptic patients, presenting with normal mucosa (NM = 53), chronic gastritis (CG = 94), and gastric cancer (GC = 82), by ELISA.

**Results:**

Moderate levels of TNF-α were detected in the NM group (19.9 ± 19.5 pg/ml), which were nearly doubled in patients with CG (35.7 ± 28.0 pg/ml) and drastically declined in GC patients (1.8 ± 5.9 pg/ml). The serum levels of IL-8, however, were not statistically different amongst these three groups.

**Conclusion:**

TNF-α serum concentration seemed to undergo up- and downregulation, when moving from NM to CG and from CG to GC, respectively. If confirmed in a prospective study, this cytokine can behave as a serum indicator of gastric inflammation and malignant transformation.

## INTRODUCTION

Gastric cancer was recognized as the fourth worldwide cause of cancer death in 2020. Among the different types of GC, the intestinal type is the most prevalent one^[^^1^^]^. This GC subtype is highly affected by environmental and genetic factors and is preceded by a cascade of precancerous lesions, as previously described by Correa and Piazuelo^[^^2^^]^. According to Correa’s model, primarily, the normal mucosa of the stomach becomes inflamed, otherwise known as gastritis. Gastric inflammation is initiated by several factors, including *H. pylori* infection, high-salt diet, nitrate intake, etc. Gastritis may then develop into atrophic gastritis, intestinal metaplasia, dysplasia, and ultimately gastric cancer^[^^2^^]^.

TNF-α is a critical cytokine, which is mainly produced by macrophages and is involved in many biological processes, such as acute and chronic inflammation. In the process of carcinogenesis, it has a dual role by which it causes tumor cell death and apoptosis, on the one hand, and potentiates tumorigenesis and progression, on the other hand^[^^3^^]^. IL-8 is another pro-inflammatory chemokine with varying sizes (reviewed in^[^^4^^]^). The activated form of IL-8 is induced in the tumor microenvironment, including cancer cells, macrophages, endothelial cells, and neutrophils and impacts several intracellular signaling cascades via binding to its G protein-coupled receptors (reviewed in^[^^4^^]^). It is involved in carcinogenesis by activating different pathways, including serine/ threonine kinases and protein tyrosine kinases and affects the transcriptome and proteome profile (reviewed in^[^^4^^]^).

While alterations in serum concentration of TNF-α and IL-8 have been investigated in several cancers, including GC, the results remain controversial, and the potential of the mentioned cytokines as serum indicators of GC is under debate. In this study, we have evaluated the serum levels of TNF-α and IL-8 and *H. pylori* serostatus in three dyspeptic groups, with varying histopathologic status, including those with NM, CG, and GC. We have also investigated the association of other modifying factors, such as age, gender, ethnicity, family history of GC with the serum levels of IL-8 and TNF-α in general and in each group separately.

## MATERIALS AND METHODS

**Study population**

The study population included histologically confirmed GC (n = 82) patients who attended Imam Khomeini Hospital, Tehran, Iran, from 2012 to 2017, as well as dyspeptic patients who were referred to the Gastroenterology Department of Amir-Alam Hospital, Tehran, Iran, at the same time period and were diagnosed as either NM (n = 53) or CG (n = 94) following histopathologic analysis. The interview questionnaire comprised of demographic characteristics, including age, gender, ethnicity, smoking habits, FHGC, and prior history of chemotherapy (Table 1). Of the 82 GC patients, 30 subjects had received chemotherapy, prior to blood sampling.

**Serum analysis**

For each patient, 2.5 ml of whole blood was collected. The sera were isolated and kept at -70°C until further analysis. A commercial anti-*H. pylori *IgG ELISA kit (SERION Diagnostics, Germany) was used to determine the *H. pylori* serostatus. To quantify IL-8 and TNF-α serum concentrations, R&D ELISA kit (DuoSet ELISA, USA) was used according to the manufacturer’s instructions.

**Statistical analysis**

The mean serum concentrations of TNF-α and IL-8 were compared amongst different patient groups, using the Kruskal-Wallis test, followed by multiple comparisons using Dunn's multiple comparisons test. Chi-square test was used for the analysis of baseline characteristics in Table 1. Data were analyzed in SPSS statistics software (version 24). Graphs were illustrated using Graphpad prism (version 8). The *p* values less than 0.05 were considered as statistically significant. 

## RESULTS AND DISCUSSION

**Study population**

Analysis of the demographic factors among the patient groups indicated that GC patients were significantly older than the non-GC (CG and NM) subjects (63 ± 11 y vs. 54 ± 11 y and 55 ± 8 y; *p* < 0.001) and were predominantly male (*p* < 0.001). Most of the GC patients were of Turk/Turkman (46%), and Kurd/Lor/Lak (26%) ethnicities, whereas in CG patients, Fars (46%) and Turk/Turkman (26%) ethnicities, and in NM patients Turk/Turkman (38%), and Fars (34%) ethnicities predominated the population. The majority of GC tumors were of the noncardia subsite (66%) and intestinal subtype (48%). 

***H. pylori*** **serostatus**

The NM group showed the lowest percentage of *H. pylori *seropositivity (32%), compared to CG (69%), and GC (54%) (*p* < 0.001). The *H. pylori* serostatus did not indicate any significant association with age, gender, ethnicity, FHGC, and smoking habits. 

**Alterations in serum TNF-α levels**

**Fig. 1:**
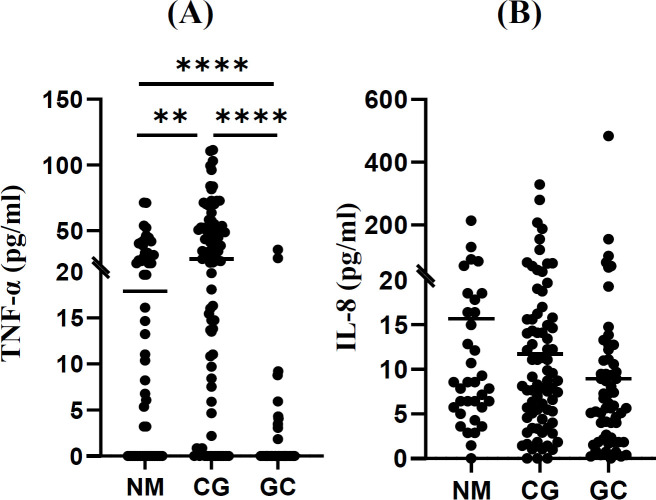
Mean serum concentrations of TNF-α (A) and IL-8 (B) in the patient groups. ^**^ and ^****^ show *p* < 0.01 and *p* < 0.0001, respectively

The serum concentration of TNF-α was significantly higher in the CG patients, as compared to the NM group (35.7 ± 28.0 vs. 19.9 ± 19.5 pg/ml; *p* = 0.0087; Fig. 1A and Table 1). TNF-α concentration was higher in GC patients, who had received chemotherapy (n = 30; 3.5 ± 6.7), as compared to those who did not (n = 53; mean ± SD: 1.3 ± 6.0 pg/ml; *p* = 0.0012). The maximum concentration of TNF-α was 71.5, 111.4, and 35.8 pg/ml, in the NM, CG, and GC groups, respectively. Combining the three groups showed that the TNF-α concentration was significantly higher in the following subjects: (1) aged <60 vs. those above (*p* = 0.001), (2) females vs. males (*p* = 0.027), (3) never vs. ever smokers (*p* = 0.009), (4) No FHGC vs. those with FHCG (*p* = 0.010), and (5) *H. pylori* seropositive vs. negative (*p* = 0.045) (Table 1). In the NM group, individuals older than 60 years of age, had significantly higher TNF-α levels, as compared to those younger (23.1 ± 20.2 vs. 9.4 ± 12.7 pg/ml; *p* = 0.033). Moreover, the TNF-α levels were not significantly different between Hp-seronegative NM and CG patients. Interestingly, the serum levels of TNF-α in CG patients were significantly lower in subjects with FHGC, as compared to those without (12.2 ± 11.4 vs. 36.8 ± 27.7; *p* = 0.003). Combining the NM and CG groups also revealed a lower TNF-α level in patients with FHGC than those without (12.5 ± 14.2 vs. 31.0 ± 36.3 pg/ml; *p* = 0.003). According to the herein performed extensive literature review (Supplementary Table 1), our results are not in line with many studies^[^^5^^–^^7^^]^ which have reported relatively higher serum levels of TNF-α in GC patients, as compared to healthy controls. However, our results are consistent with two other studies indicating a lower concentration of TNF-α in GC patients^[^^8^^,^^9^^]^. Moreover, similar to our experiment, there are several studies in which TNF-α could not be detected in the serum of all or most of the studied patients including GC and healthy individuals^[^^10^^–^^12^^]^. The absence of TNF-α production by various tumor cells, including cell lines derived from T-lymphocytes, colon and breast cancers, has also been documented^[^^13^^]^. On the other hand, in a comparative study, serum TNF-α was detectable in patients with ovarian and breast tumors, but not in GC cases^[^^14^^]^. In other studies, TNF-α serum concentrations in GC patients was similar to the controls^[^^15^^,^^16^^]^. Absence or low level of TNF-α in the serum of GC patients can have various reasons. For instance, Ohno *et al*^[^^11^^]^ have suggested that TNF-α is not always secreted, even when its mRNA is detected. These authors showed no expression of TNF-α in GC cell lines, including KATO-III and MKN-45. It has also been hypothesized that a decrease in TNF-α expression might be due to immune suppression in GC patients^[^^9^^]^. Moreover, it has been reported that the half-life of TNF-α is very short, making it difficult to detect, which leads to variable reported levels^[^^17^^]^. Another possible reason for TNF-α suppression in GC patients might be T cell exhaustion. It is well known that the effector T cells are exhausted in chronic infections and cancer and might lose their normal capacity in cytokine production^[^^18^^]^. Accordingly, TNF-α is highly suppressed in the exhausted CD8^+ ^effector T cells^[^^19^^]^. Thus, to gain more information about TNF-α expression in blood cells and tumor-infiltrating immune cells in GC patients, we analyzed the open source high-throughput data from Gene Expression Omnibus (GEO) repository at the transcript level. It should be noted that expression of TNF-α at transcript level does not necessarily reflect its expression at protein level. Supporting the T-cell exhaustion hypothesis, a recent single-cell RNA-seq of both tumor-infiltrating and peripheral blood cells of GC patients showed a marked downregulation of genes involved in the cytokine production of exhausted CD8^+ ^effector T cells^[^^20^^]^. However, TNF-α secretion is not limited to the effector T cells, but is also produced by other cell types such as macrophages and natural killer cells^[^^21^^]^. Similar to ELISA test reports on the sera of patients, the results of high-throughput analysis of immune cells in GC patients have been controversial. In the tumor-infiltrating and blood dendritic cells of GC patients, *TNF-α* mRNA expression is upregulated compared to the adjacent normal tissue and blood of healthy individuals^[^^20^^]^. Moreover, the suppression of several *TNF* receptor superfamily genes and receptor-associated factors were reported in peripheral blood leukocytes of GC patients, using RNA-seq analysis (GSE49515 dataset). In a single-cell RNA-seq analysis, the authors reported four clusters of macrophages, two of which expressed higher levels of *TNF*. At the same time, other subclusters had deficient *TNF* expression^[^^22^^]^, indicating a heterogeneous tumor microenvironment. Therefore, the inconsistent reports on TNF-α expression under cancerous conditions might be due to the heterogeneous tumor microenvironment and the functional state of the immune cells in the blood. Of note, there has been a report on the enhanced effects of chemotherapy, when combined with TNF-α treatment. In this approach, TNF-α induces apoptosis in cancer cells^[^^23^^]^. 

**Table 1:** Baseline characteristics and serum levels of IL-8 and TNF-α in the studied groups

	**Numbers (%) **				**TNF-α ** **(mean ± SD)**					**IL-8 (mean ± SD)**			
**Variables**	**GC** **(n = 82)**	**CG ** **(n = 94)**	**NM ** **(n = 53)**		**GC**	**CG**	**NM**	**All**		**GC**	**CG**	**NM**	**All**
**Serum cytokine levels**	-	-	-		1.8 ± 5.9^c^	35.7 ± 28.0 ^a^	19.9 ± 19.5 ^b^	20.1 ± 25.6		21.7± 56.8^b^	27.8 ± 51.7^a^	25.6 ± 35.9^a^	24.3 ± 49.7
													
**Age**	^b^	^a^	^a^										
< 60	31 (38)	66 (70)	41 (77)		1.0 ± 3.0^c^	35.3 ± 29.0^a^	^A^ 23.1 ± 20.2^b^	^A^24.4 ± 26.6		14.6 ± 27.9	21.1 ± 41.5	21.5 ± 26.7	19.5 ± 34.6
≥ 60	51 (64)	28 (30)	12 (23)		2.3 ± 7.1 ^c^	31.5 ± 22.9 ^a^	^B^ 9.4 ± 12.7 ^b^	^B^12.8 ± 19.9		25.5 ± 68.0	38.0 ± 65.0	39.7 ± 56.8	30.1 ± 64.6
													
**Gender**	^b^	^a^	^a^										
Female	20 (24)	52 (55)	25 (47)		3.1 ± 8.5 ^c^	36.2 ± 31.4 ^a^	17.2 ± 22.2 ^b^	^A^ 24.5 ± 28.3		15.9 ± 32.7^c^	19.1 ± 25.5^bc^	29.7 ± 30.8^a^	21.1± 28.7
Male	62 (76)	42 (45)	28 (53)		1.3 ± 4.5 ^c^	33.4 ± 24.1 ^a^	17.3 ± 21.3 ^b^	^B^ 16.8 ± 22.7		23.3 ± 62.6	37.2 ± 69.6	22.0 ± 40.1	26.6 ± 60.2
													
**Ethnicity**	^b^	^a^	^a^										
Fars	10 (12)	43 (46)	18 (34)		2.7 ± 4.4^b^	^AB^34.9 ± 26.2^a^	25.5 ± 24.5^a^	28.0 ± 26.1		11.8 ± 11.8	25.9 ± 46.1	15.1 ± 13.4	21.2 ± 37.0
Turk/Turkman	38 (46)	24 (26)	20 (38)		1.5 ± 6.3^c^	^AB^ 32.4 ± 28.8^a^	17.5 ± 14.8^b^	14.8 ± 22.1		21.3 ± 31.8	31.3 ± 65.8	33.9 ± 48.8	27.3 ± 47.8
Kurd/Lor/Lak	21 (26)	14 (15)	5 (9)		2.7 ± 7.6^b^	^AB^ 31.7 ± 25.7^a^	9.9 ± 13.6^a^	15.0 ± 21.9		33.3 ± 104.0	15.5 ± 20.0	39.4 ± 51.2	27.9 ± 77.7
Gilak/Mazani/Taleshi	10 (12)	4 (4)	7 (13)		0.9 ± 2.9	^B^ 24.7 ± 33.2	26.1 ± 19.2	13.9 ± 21.0		11.1 ± 10.3	15.7 ± 22.3	23.9 ± 23.1	16.2 ± 17.8
Other ethnicities	3 (4)	9 (10)	3 (6)		0.0^b^	^A^ 59.3 ± 30.2^a^	3.7 ± 6.4^b^	36.3 ± 37.1		12.4 ± 7.4	51.6 ± 74.8	14.9 ± 15.0	36.4 ± 60.1
													
**Smoking habits**	^b^	^a^	^b^										
Never	46 (57)	71 (75)	36 (68)		2.4 ± 7.3 ^c^	36.9 ± 29.4 ^a^	20.0 ± 17.4 ^b^	^A^ 23.2 ± 26.7		26.7 ± 74.0	27.6 ± 52.4	21.1 ± 20.7	25.1 ± 54.1
Ever	35 (43)	24 (25)	17 (32)		1.1 ± 3.3 ^c^	26.1 ± 17.9 ^a^	19.7 ± 23.8^ab^	^B^ 13.2 ± 19.2		15.5 ± 18.8	21.5 ± 41.8	35.4 ± 55.7	21.6 ± 37.5
													
**Family history of GC**													
No	70 (86)	78 (88)	44 (85)		1.7 ± 5.3 ^c^	^A^ 36.8 ± 27.7 ^a^	20.7 ± 19.7 ^b^	^A^ 21.0 ± 25.8		23.0 ± 61.3	29.0 ± 53.6	23.6 ± 35.4	25.1 ± 52.6
Yes	11 (14)	11 (12)	8 (15)		2.4 ± 8.4 ^b^	^B^ 12.2 ± 11.4 ^a^	13.1 ± 18.3 ^a^	^B^ 8.6 ± 13.1		14.0 ± 8.1	9.7 ± 10.5	35.0 ± 41.4	17.3 ± 23.7
													
***H. pylori *serostatus**	^c^	^b^	^a^										
Negative	37 (46)	29 (31)	36 (68)		1.3 ± 3.2 ^b^	30.3 ± 24.6 ^a^	18.3 ± 19.4 ^a^	^B^ 15.6 ± 21.1		18.6 ± 30.7	22.1 ± 51.4	28.0 ± 41.0	22.2 ± 40.4
Positive	44 (54)	64 (69)	17 (32)		2.0 ± 7.5 ^c^	37.1 ± 29.8 ^a^	23.5 ± 20.0 ^b^	^A^ 24.2 ± 28.4		25.5 ± 73.9	29.5 ± 51.7	20.6 ± 21.2	26.5 ± 56.9
													
**Chemotherapy**													
Yes	30 (36)	-	-		1.3 ± 6.0^B^	-	-			8.5 ± 10.3^B^	-	-	
No	40 (48)	-	-		3.5 ± 6.7^A^	-	-			28.8 ± 78.8^A^	-	-	
ND	12 (16)	-	-		0.3 ± 1.1^C^	-	-			28.7 ± 26.0^A^	-	-	
													
**Tumor subsite**													
Cardia	19 (24)	-	-		3.2 ± 4.6	-	-			11 ± 17.1	-	-	
Non-cardia	54 (66)	-	-		1.5 ± 6.6	-	-			26.8 ± 68.8	-	-	
Mixed	7 (10)	-	-		1.3 ± 3.3	-	-			12.9 ± 7.7	-	-	
IA	4 (5)	-	-		0	-	-			3.7 ± 3.8	-	-	
IB	13 (17)	-	-		4.7 ± 9.4	-	-			12.1 ± 8.4	-	-	
II	19 (25)	-	-		1.0 ± 2.5	-	-			24.8 ± 38.8	-	-	
IIIA	17 (22)	-	-		3.4 ± 9.5	-	-			39.1 ± 115.9	-	-	
IIIB	13 (17)	-	-		0	-	-			17.2 ± 19.3	-	-	
IV	10 (13)	-	-		1.7 ± 4.3	-	-			21.7 ± 20.4	-	-	
													
**Tumor grade**													
Poorly differentiated	26 (32)	-	-		2.0 ± 7.4	-	-			17.5 ± 20.6	-	-	
Moderately Differentiated	28 (34)	-	-		1.5 ± 5.8	-	-			39.2 ± 93.1	-	-	
Well-differentiated	11 (13)	-	-		0.4 ± 1.2	-	-			7.2 ± 6.5	-	-	
Unknown	17 (21)	-	-		2.6 ± 4.8	-	-			8.4 ± 10.4	-	-	
													
**Tumor subtype**													
Intestinal	24 (48)	-	-		0.9 ± 2.3	-	-			42.3 ± 100.6	-	-	
Diffuse	5 (10)	-	-		7.2 ± 16.0	-	-			16.3 ± 13.4	-	-	
Signet ring cell	11 (22)	-	-		3.8 ± 9.4	-	-			4.6 ± 4.5	-	-	
Mixed	6 (12)	-	-		2.2 ± 5.3	-	-			30.7 ± 23.6	-	-	
Other types	4 (8)	-	-		3.0 ± 1.7	-	-			8.6 ± 6.5	-	-	

For baseline characteristics, the frequencies were analyzed using chi-squared method. For categories 2-7, the frequencies were analyzed among the studied groups (GC, CG, and NM), and for categories 8-12, the frequencies were analyzed in each category in GC patients. The serum concentration of TNF-α and IL-8 were compared among the studied groups in the mentioned categories (1-11). For this comparison, in each row, variables without a common superscript letter on the right side of the numbers are statistically different (*p* < 0.05). For simplicity, the superscript letters were used only for the rows with a significant difference. Also, the concentrations were compared in each category within each studied group individually. For this comparison, in each column, variables without a common capital superscript letter on the left side of the numbers are statistically different (*p* < 0.05). For simplicity, the capital letters were used only for the columns with a significant difference.

**Alterations in serum IL-8 levels**

When the results from the three studied groups were combined, no association was found between IL-8 concentration and the listed demographic factors (Table 1). The serum IL-8 concentration among the three clinical groups (25.6 ± 35.9 [NM], 27.4 ± 51.2 [CG], and 20.2 ± 55.1 [GC]) showed that the concentration of IL-8 was significantly lower in the GC patients, as compared to the NM group (*P* = 0.027). On the other hand, in contrast to TNF-α, the serum IL-8 concentration was lower in GC patients, who received chemotherapy prior to blood sampling (8.5 ± 10.3 vs. 28.8 ± 78.8; *P* = 0.0299). Therefore, we excluded the chemotherapy-treated patients and performed a reanalysis that resulted in the elimination of the statistically significant differences among the studied groups (Fig. 1B). The maximum concentrations of IL-8 in the NM, CG, and GC groups were 213.5, 328.1, and 482.9 pg/ml, respectively. Although the serum levels of IL-8 were not significantly different among the studied groups, following the exclusion of chemotherapy-treated patients, the GC showed the highest maximum serum level of IL-8 compared to the CG and NM groups. Several studies have evaluated the levels of this cytokine in the serum of GC patients (Supplementary Table 2). In most of these studies, higher concentrations of IL-8 have been reported in the serum of GC patients compared to controls^[^^6^^,^^24^^,^^25^^]^. However, other studies have reported no significant difference between IL-8 concentration in the GC cases vs. controls^[^^15^^,^^16^^]^. The single-cell RNA-seq analysis identified a higher expression of IL-8 in the tumor infiltrating B cells of GC patients compared to the adjacent normal tissue. Nevertheless, they detected no significant differences in the IL-8 levels produced by the peripheral blood B cells from GC patients compared to healthy individuals^[^^20^^]^. Similarly, in our study, IL-8 concentrations were similar amongst the NM, CG, and GC groups. Therefore, serum IL-8 levels were unable to differentiate between the studied patient groups. 

 In conclusion, our findings indicate that serum TNF-α levels are strongly diminished in most GC patients, especially those who did not receive chemotherapy. Further analysis of the role of this cytokine in GC carcinogenesis, its regulating molecules, and its potential as a GC indicator is needed.

## DECLARATIONS

### Acknowledgments

The authors are thankful to the Pasteur Institute of Iran (Tehran) and Paris for supporting this study.

### Ethical statement

The above-mentioned sampling protocols were approved by the Ethics Committee of the Pasteur Institute of Iran, Tehran (ethical code: IR.PII.REC.1394.57). Every patient provided a written informed consent.

### Data availability

The data are available upon request.

### Author contributions

MA: carried out the study and wrote and revised manuscript; ME: provided lab assistance; MT: assisted in gastric sampling; MAM: performed gastric surgery; MEH: performed gastric endoscopy, ET: revised the manuscript; MV: revised the manuscript; MM: wrote and revised the manuscript

### Conflict of interest

None declared

### Funding/support

This project was supported by an ACIP (Pasteur International Concerted Action) grant (ACIP2015-10) from Institut Pasteur Paris; and Grant #833 from Pasteur Institute of Iran, as partial fulfillment of MA Ph.D. dissertation (code: TP-9347). 

## Supplementary Materials

Supplementary Table 1 Supplementary Table 2
