# Supplementary material for: Alteration in Serum Levels of Tumor Necrosis Factor Alpha is associated with Histopathologic Progression of Gastric Cancer
Source: Iran Biomed J. 2022 Dec 21;27(1):72–8. doi: 10.52547/ibj.3847 (PMC9971713; doi:10.52547/ibj.3847)
Supplement: Supplementary file 1 — Supplementary Table 1 [file ibj-27-72-s1.pdf]

Supplementary Table 1. Literature review of reports on serum TNF-α in GC patients

Ref, year country	Cases (n)	Control (n)	GC vs. control	Case mean level (pg/ml)	Control mean level (pg/ml)	Conclusion
[1]2007 Portugal	GC = 48	-	-	MN = 28 ± 7<br>WN = 5 ± 3	-	Malnourished patients had higher values of TNF- α.
[2]2020 Morocco	CG = 65<br>PL= 50<br>GC = 40	H = 63	GC > H	CG = 51 ± 20<br>PL= 55 ± 28<br>GC = 117 ± 92	36 ± 16	TNF-α serum levels increased in accordance with gastric lesions severity.
[3]2019 Turkey	PL = 35<br>GC = 32	H = 23	GC > H	PL= 38 ± 12<br>GC = 47 ± 15	34 ± 13	There were significant differences among patients with GC, PCL, and H in terms of TNF-α.
[4]2018 China	LG = 40<br>AOSG = 40	-	-	LG = 170 ± 51<br>AOSG = 185 ± 45	-	No significant differences between the LG and the AOSG
[5]2015 Turkey	GC = 58	H = 20	GC > H	Median = 13 (5-58)	10 (6-31)	Serum level of TNF-α may be a diagnostic marker.
[6]2003 Germany	GC = 24	H = 48	GC > H	Median = 10 (4 -46)	2 (0.2–8)	-
[7]2011 Turkey	GC = 32	H = 40	GC > H	29 ± 17	10 ± 7	Cachexia in GC is associated with changes in TNF-α.
[8]2006 Italy	GC = 10	H = 15	GC > H	205 ± 17	109 ± 9	The use of TNF-α in the screening and prognostic assessment of GC patients could be possible.
[9]1994 Japan	GC = 50	-	-	Detected in 13 out of 50 patients	-	TNF is not constantly secreted even when TNF mRNA is expressed.
[10]1995 Oman	GC = 21	H = 17	GC < H	16.9	52.34	Detectable in 6 out of 17 H<br>Detectable in 5 out of 21 GC
[11]1998 Taiwan	GC = 220	H = 85	-	Detectable in 17 out of 220 patients	Not detected	TNF-α value was not an independent prognostic indicator.
[12]2009 Poland	GC = 136	H = 42	GC > H	Detected in 52% of the patients	N/A	TNF level was significantly higher in stages I and IV of the disease.
[13]2000 Japan	GC = 20	H = 10	GC < H	107.2 ± 22.8 (45.9)	233.6 ± 48.2	-
[14]1198 Japan	GC = 12	H = 6	-	-	-	TNF-α was undetectable in the serum of both cancer patients and controls.
[15]2017 Mexico	GC = 162	H = 201	GC ~ H	-	-	No differences in TNF-α levels were observed between GC and controls.
[16]2018 China	GC = 176<br>AH = 117	H = 204	GC ~ H	AH = 6.7 (5.3, 8.3); GC = 10.2 (5.9, 16.4)	5.5 (4.2, 7.2)	-
[17]2019 China	GC = 142	H = 98	GC > H	4 (3-6)	3 (3-4)	-

WN, well-nourished; MN, malnourished; H, healthy; PL, precancerous lesions; LG, laparoscopy group; AOSG, Abdominal Open Surgery Group; AH, atypical hyperplasia; PCL, precancerous lesions

REFERENCES

1. Correia M, Cravo M, Marques-Vidal P, Grimble R, Dias-Pereira A, Faias S, Nobre-Leitão C. Serum concentrations of TNF-alpha as a surrogate marker for malnutrition and worse quality of life in patients with gastric cancer. *Clinical nutrition* 2007; **26**(6): 728-735.
2. Bounder G, Jouimyi MR, Boura H, Touati E, Michel V, Badre W, Jouhadi H, Kadi M, Eljihad M, Benomar H, Kettani A, Lebrazi H, Maachi F. Associations of the -238(G/A) and -308(G/A) TNF-α promoter polymorphisms and TNF-α serum levels with the susceptibility to castric precancerous lesions and gastric cancer related to Helicobacter pylori infection in a Moroccan population. *Asian Pacific journal of cancer prevention* 2020; **21**(6): 1623-1629.
3. Aksoy EK, Akpınar MY, Doğan Ö, Göktaş Z, Sapmaz FP, Şimşek GG, Uzman M, Nazlıgül Y. Clinical significance of serum vascular endothelial growth factor, pigment epithelium-derived factor, tumor necrosis factor alpha, and progranulin levels in patients with gastric cancer and gastric precancerous lesions. *Journal of gastrointestinal cancer* 2019; **50**(3): 537-542.
4. Zhang L, Ren L, Shan K, Guo X, Wang J, Cui B, Cui B, An J. Serum inflammatory cytokines comparison in gastric cancer therapy. *Open medicine* 2019; **14**(1): 300-306.
5. Erturk K, Tastekin D, Serilmez M, Bilgin E, Bozbey HU, Vatansever S. Clinical significance of serum interleukin-29, interleukin-32, and tumor necrosis factor alpha levels in patients with gastric cancer. *Tumour biology* 2016; **37**(1): 405-412.
6. Konturek PC, Konturek SJ, Bielanski W, Kania J, Żuchowicz M, Hartwich A, Rehfeld JF, Hahn EG. Influence of COX-2 inhibition by rofecoxib on serum and tumor progastrin and gastrin levels and expression of PPARgamma and apoptosis-related proteins in gastric cancer patients. *Digestive diseases and sciences* 2003; **48**(10): 2005-2017.
7. Kemik O, Kemik AS, Begenik H, Erdur FM, Emre H, Sumer A, Purisa S, Tuzun S, Kotanl C. The relationship among acute-phase responce proteins, cytokines, and hormones in various gastrointestinal cancer types patients with cachectic. *Human and experimental toxicology* 2012; **31**(2): 117-125.
8. Macrì A, Versaci A, Loddo S, Scuderi G, Travagliante M, Trimarchi G, Teti D, Famulari C. Serum levels of interleukin 1beta, interleukin 8 and tumour necrosis factor alpha as markers of gastric cancer. *Biomarkers* 2006; **11**(2): 184-193.
9. Ohno M, Kato M, Nakamura T, Saitoh Y. Gene expression for tumor necrosis factor alpha and its production in gastric cancer patients. *Japanese journal of cancer research* 1994; **85**(10): 1029-1034.
10. Kabir S, Grant C, Daar AS. Serum levels of interleukin-1, interleukin-6 and tumour necrosis factor-alpha in patients with gastric carcinoma. *Cancer letters* 1995; **95**(1-2): 207-212.
11. Wu CW, Chi CW, Hsieh MC, Chao MF, Lui WY, P'Eng FK. Serum tumor necrosis factor in patients with gastric cancer. *Anticancer research* 1998; **18**: 1597-1599.
12. Szaflarska A, Szczepanik A, Siedlar M, Czupryna A, Sierzega M, Popiela T, Zembala M. Preoperative plasma level of IL-10 but not of proinflammatory cytokines is an independent prognostic factor in patients with gastric cancer. *Anticancer research* 2009; **29**(12): 5005-5012.
13. Shimamura H, Iwagaki H, Gouchi A, Morimoto Y, Ariki N, Funaki M, Tanaka N. Autologous serum deprivation restored IL-1 receptor antagonist production by peripheral blood mononuclear cells in patients with gastric cancer. *The journal of international medical research* 2000; **28**(6): 277-287.
14. Makino T, Noguchi Y, Yoshikawa T, Doi C, Nomura K. Circulating interleukin 6 concentrations and insulin resistance in patients with cancer. *The British journal of surgery* 1998; **85**(12): 1658-1662.
15. Sánchez-Zauco N, Torres J, Gómez A, Camorlinga-Ponce M, Muñoz-Pérez L, Herrera-Goepfert R, Medrano-Guzmán R, Giono-Cerezo S, Maldonado-Bernal C. Circulating blood levels of IL-6, IFN-γ, and IL-10 as potential diagnostic biomarkers in gastric cancer: a controlled study. *BMC cancer* 2017; **17**(1): 384.
16. Li J, Xu L, Run ZC, Feng W, Liu W, Zhang PJ, Li Z. Multiple cytokine profiling in serum for early detection of gastric cancer. *World journal of gastroenterology* 2018; **24**(21): 2269-2278.
17. Sun X, Xiang C, Wu J, Dong W, Zhan Z, Wang R, Zhang JF. Relationship between serum inflammatory cytokines and lifestyle factors in gastric cancer. *Molecular and clinical oncology* 2019; **10**(3): 401-414.
