# Supplementary material for: Alteration in Serum Levels of Tumor Necrosis Factor Alpha is associated with Histopathologic Progression of Gastric Cancer
Source: Iran Biomed J. 2022 Dec 21;27(1):72–8. doi: 10.52547/ibj.3847 (PMC9971713; doi:10.52547/ibj.3847)
Supplement: Supplementary file 2 — Supplementary Table 2 [file ibj-27-72-s2.pdf]

Supplementary Table 2. Literature review of reports on serum IL-8 in GC patients

Ref, year country	Cases (n)	Control (n)	GC vs. control	case level mean/ median (pg/ml)	Control level mean/ median (pg/ml)	Conclusion
[16]2018 China	GC = 176<br>AH = 117	H = 204	GC ~ H	AH = 0.9 (0.3, 2)<br>GC= 2 (0.1, 6)	0.5 (0.1, 1)	-
[18]2002 Poland	GC = 337	H = 337	GC > H	-	-	Serum IL-8 values over the cut-off point (1.77 pg/mL) occurred in almost all (99.7%) GC patients but in only a few controls (0.3%).
[19]2015 India	FD = 60<br>GC= 60	H=60	GC > H	FD: 54 (4-319)<br>GC: 58 (6-320)	26 (5-305)	-
[7]2011 Turkey	GC = 32	H = 40	GC > H	83.1 + 19.6	29.5 + 19.6	-
[20]2009 China	GC = 125	H = 140	GC > H	1178 ± 84	341 ± 17	-
[15]2017 Mexico	GC = 162	H = 201	GC ~ H	-	-	No differences in IL-8 levels were observed between GC and controls.
[21]2014 Mexico	GC = 123	NAG = 54<br>H = 48	GC > NAG, H	48	NAG = 12<br>H = 21	-
[17]2019 China	GC = 142	H = 98	GC > H	105 (48-260)	42 (24-111)	-
[8]2006 Italy	GC = 10	H = 15	GC > H	283 ± 28	137 ± 7	-

AH, atypical hyperplasia; FD, functional dyspepsia; H, health

REFERENCES

1. Correia M, Cravo M, Marques-Vidal P, Grimble R, Dias-Pereira A, Faias S, Nobre-Leitão C. Serum concentrations of TNF-alpha as a surrogate marker for malnutrition and worse quality of life in patients with gastric cancer. *Clinical nutrition* 2007; **26**(6): 728-735.
2. Bounder G, Jouimyi MR, Boura H, Touati E, Michel V, Badre W, Jouhadi H, Kadi M, Eljihad M, Benomar H, Kettani A, Lebrazi H, Maachi F. Associations of the -238(G/A) and -308(G/A) TNF-α promoter polymorphisms and TNF-α serum levels with the susceptibility to castric precancerous lesions and gastric cancer related to Helicobacter pylori infection in a Moroccan population. *Asian Pacific journal of cancer prevention* 2020; **21**(6): 1623-1629.
3. Aksoy EK, Akpınar MY, Doğan Ö, Göktaş Z, Sapmaz FP, Şimşek GG, Uzman M, Nazlıgül Y. Clinical significance of serum vascular endothelial growth factor, pigment epithelium-derived factor, tumor necrosis factor alpha, and progranulin levels in patients with gastric cancer and gastric precancerous lesions. *Journal of gastrointestinal cancer* 2019; **50**(3): 537-542.
4. Zhang L, Ren L, Shan K, Guo X, Wang J, Cui B, Cui B, An J. Serum inflammatory cytokines comparison in gastric cancer therapy. *Open medicine* 2019; **14**(1): 300-306.
5. Erturk K, Tastekin D, Serilmez M, Bilgin E, Bozbey HU, Vatansever S. Clinical significance of serum interleukin-29, interleukin-32, and tumor necrosis factor alpha levels in patients with gastric cancer. *Tumour biology* 2016; **37**(1): 405-412.
6. Konturek PC, Konturek SJ, Bielanski W, Kania J, Żuchowicz M, Hartwich A, Rehfeld JF, Hahn EG. Influence of COX-2 inhibition by rofecoxib on serum and tumor progastrin and gastrin levels and expression of PPARgamma and apoptosis-related proteins in gastric cancer patients. *Digestive diseases and sciences* 2003; **48**(10): 2005-2017.
7. Kemik O, Kemik AS, Begenik H, Erdur FM, Emre H, Sumer A, Purisa S, Tuzun S, Kotanl C. The relationship among acute-phase responce proteins, cytokines, and hormones in various gastrointestinal cancer types patients with cachectic. *Human and experimental toxicology* 2012; **31**(2): 117-125.
8. Macrì A, Versaci A, Loddo S, Scuderi G, Travagliante M, Trimarchi G, Teti D, Famulari C. Serum levels of interleukin 1beta, interleukin 8 and tumour necrosis factor alpha as markers of gastric cancer. *Biomarkers* 2006; **11**(2): 184-193.
9. Ohno M, Kato M, Nakamura T, Saitoh Y. Gene expression for tumor necrosis factor alpha and its production in gastric cancer patients. *Japanese journal of cancer research* 1994; **85**(10): 1029-1034.
10. Kabir S, Grant C, Daar AS. Serum levels of interleukin-1, interleukin-6 and tumour necrosis factor-alpha in patients with gastric carcinoma. *Cancer letters* 1995; **95**(1-2): 207-212.
11. Wu CW, Chi CW, Hsieh MC, Chao MF, Lui WY, P'Eng FK. Serum tumor necrosis factor in patients with gastric cancer. *Anticancer research* 1998; **18**: 1597-1599.
12. Szaflarska A, Szczepanik A, Siedlar M, Czupryna A, Sierzega M, Popiela T, Zembala M. Preoperative plasma level of IL-10 but not of proinflammatory cytokines is an independent prognostic factor in patients with gastric cancer. *Anticancer research* 2009; **29**(12): 5005-5012.
13. Shimamura H, Iwagaki H, Gouchi A, Morimoto Y, Ariki N, Funaki M, Tanaka N. Autologous serum deprivation restored IL-1 receptor antagonist production by peripheral blood mononuclear cells in patients with gastric cancer. *The journal of international medical research* 2000; **28**(6): 277-287
14. Makino T, Noguchi Y, Yoshikawa T, Doi C, Nomura K. Circulating interleukin 6 concentrations and insulin resistance in patients with cancer. *The British journal of surgery* 1998; **85**(12): 1658-1662.
15. Sánchez-Zauco N, Torres J, Gómez A, Camorlinga-Ponce M, Muñoz-Pérez L, Herrera-Goepfert R, Medrano-Guzmán R, Giono-Cerezo S, Maldonado-Bernal C. Circulating blood levels of IL-6, IFN-γ, and IL-10 as potential diagnostic biomarkers in gastric cancer: a controlled study. *BMC cancer* 2017; **17**(1): 384.
16. Li J, Xu L, Run ZC, Feng W, Liu W, Zhang PJ, Li Z. Multiple cytokine profiling in serum for early detection of gastric cancer. *World journal of gastroenterology* 2018; **24**(21): 2269-2278.
17. Sun X, Xiang C, Wu J, Dong W, Zhan Z, Wang R, Zhang JF. Relationship between serum inflammatory cytokines and lifestyle factors in gastric cancer. *Molecular and clinical oncology* 2019; **10**(3): 401-414.
18. Konturek SJ, Starzynska T, Konturek PC, Karczewska E, Marlicz K, Lawniczak M, Jaroszewicz-Heigelman H, Bielanski W, Hartwich A, Ziemniak A, Hahn E G. Helicobacter pylori and CagA status, serum gastrin, interleukin-8 and gastric acid secretion in gastric cancer. *Scandinavian journal of gastroenterology* 2002; **37**(8): 891-898.
19. Kumar S, Kumari N, Mittal RD, Mohindra S, Ghoshal UC. Association between pro-(IL-8) and anti-inflammatory (IL-10) cytokine variants and their serum levels and H. pylori-related gastric carcinogenesis in northern India. *Meta gene* 2015; **6**: 9-16.
20. Song, BoZhang D, Wang S, Zheng H, Wang X. Association of interleukin-8 with cachexia from patients with low-third gastric cancer. *Comparative and functional genomics* 2009; **2009**: 212345.
21. Fernandes J, Michel V, Camorlinga-Ponce M, Gomez A, Maldonado C, De Reuse H, Torres J, Touati E. Circulating mitochondrial DNA level, a noninvasive biomarker for the early detection of gastric cancer. *Cancer epidemiology, biomarkers and prevention* 2014; **23**(11): 2430-2438.
